# Haemotherapy with Fibrinogen for Perioperative Bleeding Prevention—A View on Arterial Thrombogenesis and Myocardial Infarction in the Rat In Vivo

**DOI:** 10.3390/jcm8060880

**Published:** 2019-06-19

**Authors:** André Heinen, Vera Welke, Friederike Behmenburg, Martin Stroethoff, Volker Stoldt, Till Hoffmann, Markus W. Hollmann, Ragnar Huhn

**Affiliations:** 1 Institute of Cardiovascular Physiology, Heinrich-Heine-University Duesseldorf, Universitaetsstr. 1, 40225 Duesseldorf, Germany; Andre.Heinen@uni-duesseldorf.de; 2 Department of Anesthesiology, University Hospital Duesseldorf, Moorenstr. 5, 40225 Duesseldorf, Germany; vera8383@aol.com (V.W.); Friederike@Behmenburg.de (F.B.); Martin.Stroethoff@med.uni-duesseldorf.de (M.S.); 3 Department of General, Visceral and Pediatric Surgery, University Hospital Duesseldorf, Moorenstr. 5, 40225 Duesseldorf, Germany; Volker.Stoldt@med.uni-duesseldorf.de; 4 Institute of Transplantation Diagnostics and Cell Therapeutics, University Hospital Duesseldorf, Moorenstr. 5, 40225 Duesseldorf, Germany; Till.Hoffmann@med.uni-duesseldorf.de; 5 Department of Anesthesiology, Amsterdam University Medical Center (AUMC), Location AMC, Meiberdreef 9, 1105 AZ Amsterdam, The Netherlands; M.W.Hollmann@amc.uva.nl

**Keywords:** coagulation, cardioprotection, thrombosis

## Abstract

Major blood loss during cardiac surgery is associated with increased morbidity and mortality. Clinical pilot studies indicated that preoperative fibrinogen supplementation reduces postoperative blood loss without increasing thrombotic complications. However, an increase in fibrinogen concentration might rather aggravate pre-existing thrombosis than increase the incidence of thrombotic events. Therefore, we investigated, in the present study, whether fibrinogen supplementation influences (1) arterial thrombus formation, (2) the extent of myocardial infarction and (3) the cardioprotective effect of ischaemic preconditioning. Arterial thrombogenesis of the femoral artery was induced by topic FeCl_3_ treatment in anaesthetised Wistar rats after pretreatment with 60 mg/kg (Fib_low_), 120 mg/kg (Fib_high_) or vehicle (Con). Vessel blood flow was monitored, and time to vessel occlusion was analysed as a marker for arterial thrombogenesis. In addition, regional myocardial I/R injury was induced by temporary left coronary artery occlusion in rats pretreated with or without fibrinogen supplementation. In additional groups, ischaemic preconditioning (IPC) was induced by 3 cycles of 5 min of ischaemia/reperfusion. In all groups, myocardial infarct size was determined by triphenyltetrazoliumchlorid staining. Arterial thrombogenesis was not affected by fibrinogen pretreatment. No differences in time until vessel occlusion between Con, Fib_low_ and Fib_high_ groups were observed. In addition, fibrinogen supplementation in low and high concentrations had no effect on infarct size after regional myocardial ischaemia and reperfusion (Fib_low_: 66 ± 10%, Fib_high_: 62 ± 9%; each ns vs. Con). IPC reduced infarct size from 62 ± 14% to 34 ± 12% (*p* < 0.05 vs. Con). Furthermore, both fibrinogen concentrations did not affect cardioprotection by ischaemic preconditioning (Fib_low_ + IPC: 34 ± 11%, Fib_high_ + IPC: 31 ± 13%; each ns vs. IPC). Haemotherapy with fibrinogen did not affect arterial thrombogenesis, myocardial infarction and the cardioprotective effect of ischaemic preconditioning.

## 1. Introduction

Severe bleeding during cardiac surgery is associated with increased morbidity and mortality [1,2]. Therefore, prophylaxis of perioperative bleeding is of great importance. However, therapeutic strategies to improve haemostasis may cause adverse events and have a significant economic impact. Perioperative bleeding is caused by both surgical trauma as well as impaired haemostasis. Although previous studies have shown an inverse correlation between preoperative fibrinogen levels and the extent of bleeding after coronary artery bypass grafting (CABG) [3,4,5,6,7], a recent meta-analysis found only a weak-to-moderate correlation between fibrinogen level and postoperative blood loss [8]. Here, the discrepancy in results from the clinical studies is discussed in detail, and the authors suggest that further clinical trials are required before making general recommendations on treatment. However, according to recent pilot studies, fibrinogen supplementation might have preventive capacity with respect to perioperative bleeding. Postoperative bleeding after CABG is reduced by employing prophylactic haemotherapy with fibrinogen [9,10]. On the other hand, therapeutic interventions leading to a prothrombotic shift of the haemostatic balance bears the risk of thrombotic and ischaemic complications. In particular, patients with pre-existing ischaemic diseases such as perioperative myocardial infarction might be at risk following fibrinogen supplementation. Perioperative elevation in fibrinogen concentration may delay or diminish reperfusion of the ischaemic myocardium by extended thrombus formation due to increased coagulation or platelet linkage. Furthermore, prophylactic treatment with fibrinogen could affect cardioprotection by ischaemic preconditioning (IPC), a setting in which the myocardium is protected against deleterious consequences of prolonged ischaemia and reperfusion by preceding short periods of myocardial ischaemia [11]. IPC causes a significant reduction in platelet activation by diminished fibrinogen retention [12], suggesting a direct mechanistic link between IPC and the interaction of fibrinogen with platelets in the cardioprotective mechanism of IPC. Here, therapeutic interventions to elevate the fibrinogen plasma concentration might counteract the anti-aggregatory effects of IPC, resulting in an attenuation of its cardioprotective potential. Therefore, we hypothesised that haemotherapy with fibrinogen in the rat in vivo (1) promotes arterial thrombus formation and stability, (2) increases myocardial infarct size and (3) counteracts the cardioprotective potency of ischaemic preconditioning.

## 2. Material and Methods

The current investigation was conducted in accordance with the Guide for the Care and Use of Laboratory Animals published by the National Institutes of Health (Publication number 85-23, revised 1996) and was performed after obtaining approval from the Animal Ethics Committee of the University of Düsseldorf, Germany. The experiments were performed and the results are reported in accordance with the ARRIVE guidelines. Male Wistar rats (Janvier Laboratories, Le Genest-Saint-Isle, France), weighing 250–300 g, were housed on a 12:12 light/dark schedule with free access to standard chow and water. All chemicals were purchased from Sigma-Aldrich (Taufkirchen, Germany).

### 2.1. Pilot Experiments

**Figure 1:**
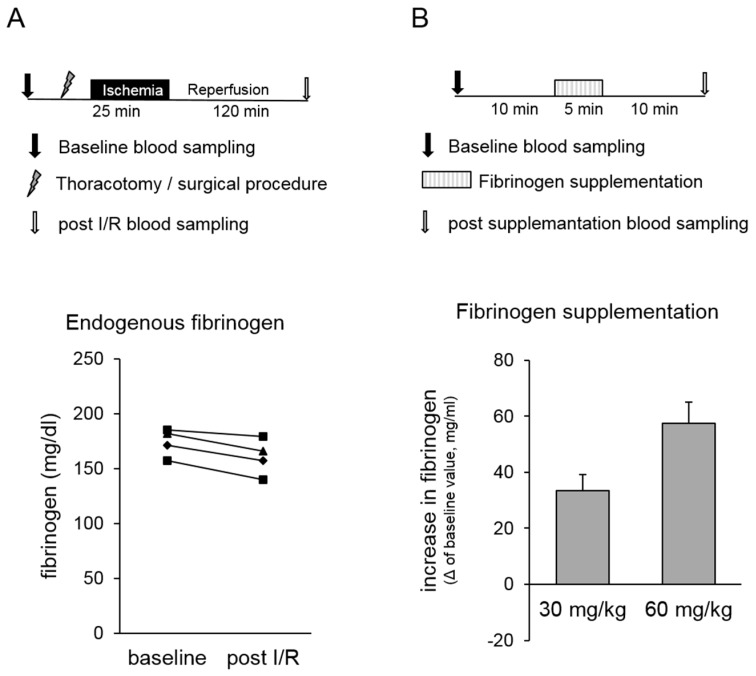
Pilot experiments. (**A**) Effect of the surgical procedure and myocardial infarction on endogenous fibrinogen levels. The upper panel shows a schematic diagram of the experimental protocol for myocardial ischaemia/reperfusion (I/R) injury. Blood was collected before (baseline) and after the surgical procedure and I/R injury (post-I/R). The lower panel illustrates the time course of fibrinogen levels of four individual animals. (**B**) Effect of fibrinogen supplementation on fibrinogen levels. The upper panel represents the experimental procedure. Rats received 30 mg/kg or 60 mg/kg fibrinogen as continuous infusion over 5 min. Blood was collected 10 min before the start (baseline) and 10 min after the end of fibrinogen infusion. Supplementation effect is shown as increase in fibrinogen level compared to baseline value (lower panel).

We conducted pilot experiments to answer the following questions with regard to the planned experimental study:

1. In our in vivo rat model, both the surgical procedure and myocardial infarction might cause a change in “endogenous” fibrinogen concentration that possibly influenced our study outcome. Therefore, pilot experiments were conducted, and fibrinogen concentrations were analysed before and after surgical procedure, including thoracotomy and myocardial ischaemia/reperfusion (I/R) injury (Figure 1A, upper panel). Methodological details are described below (please see In vivo model of myocardial infarction and ischaemic preconditioning).
2. Postoperative blood loss after coronary artery bypass grafting was significantly reduced by preoperative substitution of 2 g fibrinogen, causing an increase in plasma fibrinogen of ~60 mg/dL [9]. Therefore, we conducted pilot experiments to determine the required amount of fibrinogen yielding at an increase in fibrinogen concentration of ~60 mg/dL in our in vivo rat model (Figure 1B, upper panel). For this, pentobarbital anaesthetised rats (bolus 100 mg/kg BW i.p., continuously 40 mg/kg/h i.v.) received 30 mg/kg or 60 mg/kg fibrinogen in 0.9% NaCl as continuous infusion over 5 min via a catheter that was placed in the right jugular vein. Blood was collected 10 min before the start (baseline) and 10 min after the end of fibrinogen infusion via a catheter that was placed in the left carotid artery.

### 2.2. In Vivo Model of Thrombus Formation

**Figure 2:**
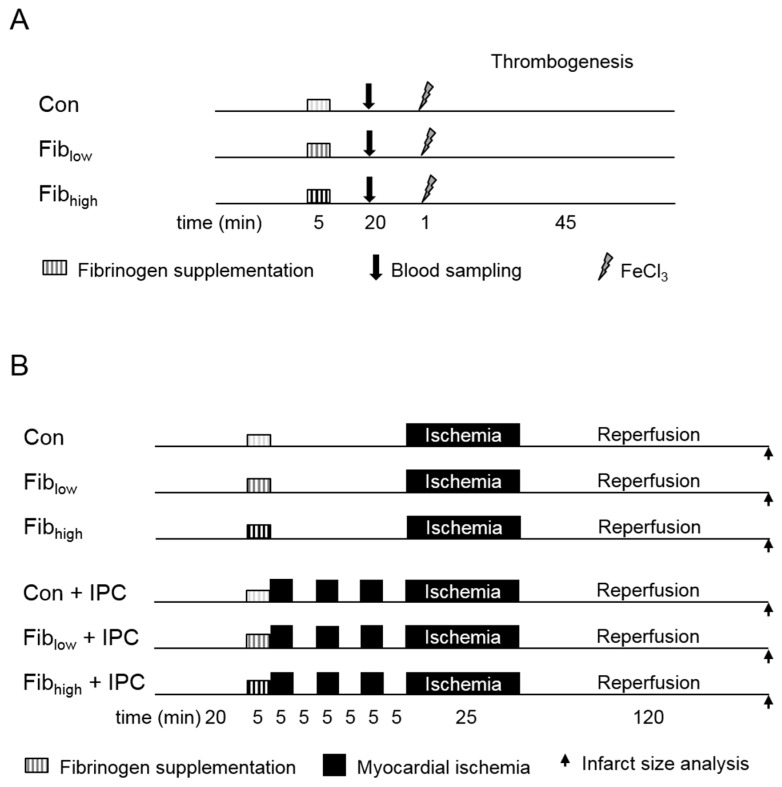
Schematic diagram of the experimental protocol for (A) arterial thrombus formation and (B) myocardial I/R injury. (**A**) Anaesthetised rats received vehicle (Con), 60 mg/kg (Fib_low_) or 120 mg/kg fibrinogen (Fib_high_) 20 min before FeCl_3_-induced thrombosis of the femoral artery. Arrows indicate the time point of blood sampling. (**B**) I/R injury was induced after pretreatment with vehicle (Con), 60 mg/kg (Fib_low_) or 120 mg/kg fibrinogen (Fib_high_) by 25 min of left coronary artery (LCA) occlusion followed by 120 min of reperfusion. In additional groups, ischaemic preconditioning (IPC) was induced by three periods of 5 min of myocardial ischaemia each followed by 5 min of reperfusion. Arrows indicate the end of reperfusion and infarct size staining.

**Figure 3:**
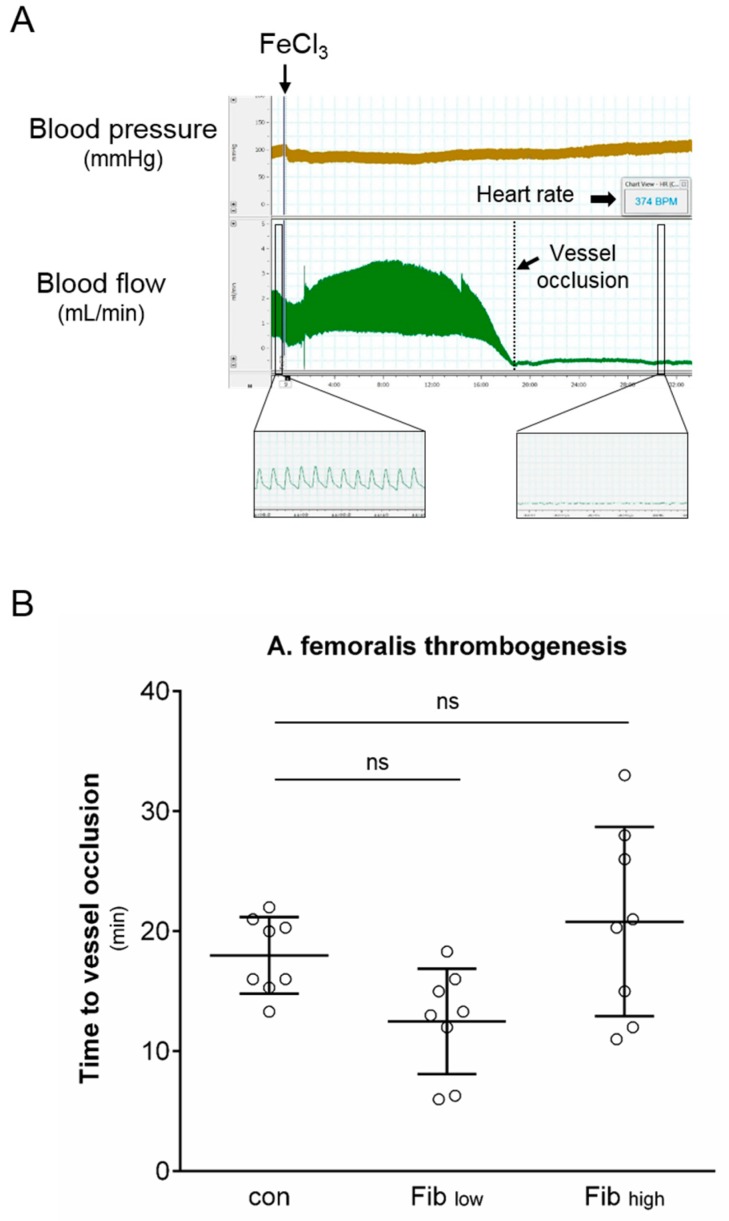
Effect of fibrinogen substitution on arterial thrombogenesis. (**A**) An example experiment is shown to illustrate the experimental procedure. Arterial blood pressure and blood flow of the femoral artery were monitored continuously. Femoral artery thrombosis was induced by FeCl_3_, and the time to complete vessel occlusion was detected. (**B**) Summarised data for arterial thrombogenesis of experimental groups pretreated with vehicle (Con), 60 mg/kg (Fib_low_) or 120 mg/kg fibrinogen (Fib_high_). Data are presented as mean ± SD, *n* = 8 for all groups. (One-way ANOVA followed by Tukey’s post hoc test). ns = not significant.

In male Wistar rats, anaesthesia was induced and maintained by administration of pentobarbital (bolus 100 mg/kg BW i.p., continuously 40 mg/kg/h i.v.). After endotracheal intubation, the right jugular vein was cannulated for administration of anaesthetics and fibrinogen, and the left carotid artery was cannulated for measurement of aortic pressure. ECG, arterial pressure and heart rate were continuously monitored and recorded on a personal computer (Chart for Windows; ADInstruments Pty Ltd, Castle Hill, Lexington, Australia) at a sampling rate of 500 Hz using an analogue-to-digital converter (PowerLab/8SP, ADInstruments Pty Ltd, Castle Hill, Lexington, Australia). After surgical preparation of the femoral artery, an ultrasonic transducer was placed around the femoral artery for blood flow measurement. Application of iron (III) chloride (FeCl_3_, 12%) on the vessel for 60 s induced a lesion with subsequent thrombosis (Figure 2A and Figure 3A). Blood flow was measured continuously, and time was detected until complete vascular obliteration as well as the time until possible vessel reopening [13].

Rats were randomised to one of the following experimental groups (Figure 2A): (1) Con: FeCl_3_ induced vessel lesion with following thrombosis, (2) Fib_low_: Fibrinogen administration (60 mg/kg BW i.v.) before FeCl_3_ induced vessel lesion with following thrombosis and (3) Fib_high_: Fibrinogen administration (120 mg/kg BW i.v.) before FeCl_3_ induced vessel lesion with following thrombosis. Fibrinogen was administered as continuous infusion over 5 min starting 20 min before the initiation of thrombogenesis by FeCl_3_. Fibrinogen was dissolved in 0.9% saline; animals of the control groups received saline.

### 2.3. In Vivo Model for Myocardial Infarction and Ischaemic Preconditioning

Anaesthesia, endotracheal intubation and cannulation of the vessels were performed as described above. Surgical preparation for temporary occlusion of the left coronary artery (LCA) was performed as described previously [14]. Here, a lateral left-sided thoracotomy was performed, and a ligature (5-0 Prolene) was passed below the LCA. All animals recovered for 20 min before randomisation and the start of the respective experimental protocol (Figure 2B). Fibrinogen was administered as continuous infusion over 5 min starting 35 min before the prolonged ischaemia in a low (Fib_low_, 60 mg/kg BW) or a high concentration (Fib_high_, 120 mg/kg BW). Control animals received saline. IPC was induced by 3 cycles of 5 min myocardial ischaemia and reperfusion prior to 25 min of ischaemia and 120 min of reperfusion. The duration of ischaemia was chosen based on our experience that this protocol results in an expected infarct size in nontreated control rats of about 55–60% of the left ventricle, i.e., values that allow the detection of both protective and adverse effects on infarct size. At the end of the experiments, hearts were excised with the occluding suture left in place and mounted on a modified Langendorff apparatus for perfusion with ice-cold normal saline via the aortic root at a perfusion pressure of 80 cm H_2_O in order to wash out intravascular blood. After 5 min of perfusion, the coronary artery was re-occluded, and the non-occluded coronary arteries were perfused through the aortic root with 0.2% Evans blue in normal saline for 10 min. Intravascular Evans blue was then washed out by perfusion with normal saline for 10 min. This treatment identified the area at risk as unstained. The heart was cut into 2 mm thick transverse slices. The slices were stained with 0.75% triphenyltetrazolium chloride solution for 10 min at 37 °C and fixed in 4% formalin solution for 24 h at room temperature. The area of risk and the infarcted area were determined by planimetry using SigmaScan Pro 5® computer software (SPSS Science Software, Chicago, IL, USA).

### 2.4. Statistical Analysis

Data are presented as mean ± SD. For thrombus formation as well as myocardial infarction experiments, the sample sizes were calculated a priori using the ANOVA sample size analysis software tool of SigmaPlot 13 (Systat Software, San Jose, CA, USA). This calculation resulted in a required group size of *n* = 8 (power: 0.8; alpha: 0.05; estimated relevant effect size: 0.30; estimated SD: 0.17) in thrombus formation experiments, and a required group size of *n* = 6 for infarct size experiments (power 0.8; alpha 0.05; estimated relevant effect size: 0.25; estimated SD: 0.10). For infarct sizes and thrombus formation, differences between groups were analysed by one-way ANOVA with Tukey post hoc test. Comparisons of haemodynamics between groups or between different time points within a group were performed with a two-way ANOVA followed by Tukey’s post hoc test. Statistical data analysis was performed using GraphPad StatMate™ Version 1.01 (GraphPad Software, San Diego, CA, USA) or SigmaPlot 13 (Systat Software, San Jose, CA, USA). Differences were considered statistically significant when *p* values were less than 0.05.

## 3. Results

### 3.1. Pilot Experiments

1. Fibrinogen levels were determined at baseline and after the surgical procedure, including thoracotomy and myocardial ischaemia/reperfusion injury in rats in vivo. No changes in endogenous fibrinogen concentration were observed within the time window that is relevant for our study protocol (Figure 1A, lower panel).
2. The required amount of fibrinogen leading to an increase of fibrinogen in plasma of ~60 mg/dL was determined. This substitution corresponds to an intraoperative substitution of 2 g fibrinogen in humans [9]. Our results show that a substitution of 60 mg/kg body weight was required to increase the fibrinogen concentration by approximately 60 mg/dL in the rat (Figure 1B, lower panel). Therefore, all further experiments were conducted after supplementation of 60 mg/kg body weight fibrinogen (Fib_low_). In addition, groups with animals receiving higher fibrinogen doses of 120 mg/kg body weight Fibrinogen (Fib_high_) were performed in the arterial thrombus formation experiments as well as in the myocardial ischaemia and reperfusion experiments to test the potential occurrence of prothrombotic side-effects limiting the therapeutic window of fibrinogen supplementation.

### 3.2. In Vivo Model of Thrombus Formation

Thrombus formation was induced by FeCl_3_ lesion of the femoral artery (Figure 3A). Blood flow was measured continuously by an ultrasonic transducer, which was placed around the vessel. Time was detected until complete vascular obliteration as well as the time until possible reopening. There were no differences in time to thrombosis between experimental groups (Con: 18 ± 3 min, Fib_low_: 13 ± 4 min, Fib_high_: 21 ± 8 min, n.s., each group *n* = 8, Figure 3B), and there was no vascular reopening in either group during the time window that was relevant for our study protocol.

#### Coagulation Parameter and Haemodynamic Data

Fibrinogen supplementation resulted in an increase in blood fibrinogen levels in both the Fib_low_ and the Fib_high_ group compared to control group (Table 1). In addition, no differences with respect to INR, aPTT or platelet count were detected between experimental groups (Table 1).

The analysis of haemodynamic variables showed no differences in heart rate, mean aortic pressure or blood flow between groups before the induction of femoral artery thrombus formation (Table 2).

### 3.3. In Vivo Model for Myocardial Infarction and Ischaemic Preconditioning

**Figure 4:**
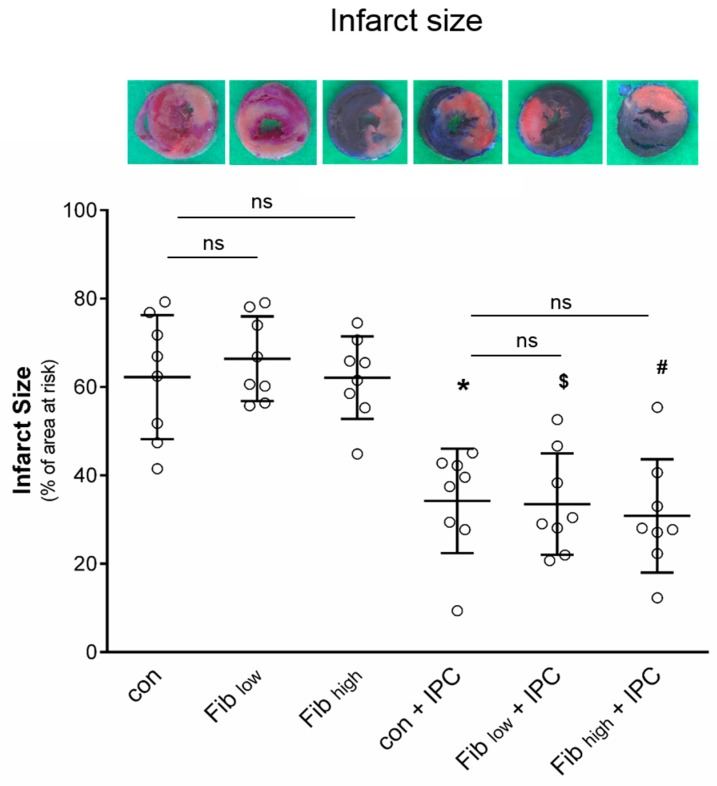
Effect of fibrinogen substitution on infarct size. Example heart slices after Evan’s Blue/TTC staining are shown for all groups (blue = remote myocardium, red = viable myocardium within the area at risk, pale = infarction), and the histogram shows the infarct size in percent of the left ventricle. Animals received either fibrinogen (60 mg/kg, Fib_low_ or 120 mg/kg, Fib_high_) or saline (Con) alone or in combination with ischaemic preconditioning (IPC). Data are presented as mean±SD, *n* = 8 for all groups. (One-way ANOVA followed by Tukey’s post hoc test). * *p* < 0.05 vs Con. ^$^ *p* < 0.05 vs Con (Fib_low_). ^#^ *p* < 0.05 vs Con (Fib_high_). ns = not significant.

Occlusion of the LCA for 25 min followed by 120 min reperfusion caused an infarct size of 62 ± 14% of the area at risk in the control group. Fibrinogen supplementation before I/R injury had no effect on infarct size compared to control group, neither in the low (Fib_low_: 66 ± 10%, ns vs. Con) nor in the high concentration (Fib_high_: 62 ± 9%; ns vs. Con, Figure 4) group.

Ischaemic preconditioning reduced infarct size by ~45% compared to the nonsupplemented control group (Con + IPC: 34 ± 12%, *p* < 0.05 vs. Con). Furthermore, neither the low nor the high fibrinogen concentration affected the cardioprotective potential of ischaemic preconditioning (Fib_low_ + IPC: 34 ± 11%, Fib_high_ + IPC: 31 ± 13%; each ns vs. IPC, Figure 4). 

In addition, the areas at risk were not different between experimental groups, indicating comparable occlusion regions in all experiments (Table 3, upper panel).

#### Haemodynamic Data

Haemodynamic variables are summarised in Table 3. Compared to baseline, mean aortic pressure was decreased in all groups with the exception of the control group at the end of the experiments. No differences in heart rate and mean aortic pressure were observed between the experimental groups during baseline, ischaemia and the reperfusion period.

## 4. Discussion

This study examined potential prothrombotic side-effects of fibrinogen supplementation on arterial thrombus formation, myocardial I/R injury, and the efficacy of cardioprotection by IPC in the rat in vivo. As fibrinogen supplementation did not influence arterial thrombogenesis, myocardial infarct size after I/R or cardioprotection by IPC, the results suggest that fibrinogen at the doses tested and in the setting described does not bear an increased risk of prothrombotic adverse effects. The absence of those adverse effects is essential for the clinical potential of prophylactic fibrinogen therapy to reduce perioperative blood loss.

Fibrinogen is a 340 kDa glycoprotein synthesised in the liver. It is critically involved in haemostasis and the first coagulation factor to reach a critical level in major bleeding [15,16]. During massive blood loss, its production becomes inadequate to compensate for the deficit [16,17]. Thus, fibrinogen supplementation to restore plasma fibrinogen is key to normalising clotting function [18,19,20]. Haemotherapy with fibrinogen is widely used in cardiac surgery. The guidelines of the European Society of Anaesthesiology on management of severe bleeding recommend in complex cardiovascular surgery fibrinogen concentrate infusion guided by viscoelastic haemostatic assay monitoring to reduce perioperative blood loss (Grade 1B) [21]. In addition, there is some evidence that preoperative fibrinogen administration before cardiac surgery reduces postoperative bleeding and maintains postoperative haemoglobin levels at a higher level [9]. In this study, a prophylactic preoperative infusion of 2 g fibrinogen increased plasma fibrinogen concentration by ~60 mg/dL [9]. Our data indicate that 60 mg/kg BW fibrinogen concentrate infusion in the rat in vivo resulted in a comparable increase in plasma fibrinogen concentration. Therefore, this dose was chosen as “low” fibrinogen dose for the following experiments investigating potential adverse effects of fibrinogen supplementation on arterial thrombogenesis, myocardial ischaemia/reperfusion injury and cardioprotection by ischaemic preconditioning.

### 4.1. Fibrinogen, Thrombogenesis and Myocardial Infarction

Infusion of coagulation factors may induce a state of hypercoagulability with an increased risk of thromboembolic events and thus may result in delayed or diminished reperfusion of the ischaemic myocardium in cardiac surgery by extended thrombus formation. Epidemiologic studies have documented an association of elevated plasma fibrinogen levels with ischaemic heart disease [22,23]. Elevated baseline serum fibrinogen levels were reported to be an independent risk factor for periprocedural myocardial infarction in patients undergoing elective percutaneous coronary intervention (PCI) [24]. Additionally, an elevated fibrinogen level (≥280 mg/dL) was reported to be associated with major adverse cardiovascular events—i.e., acute coronary syndrome—6 months after PCI [25]. To date, the underlying cause for the described adverse effects remains unknown [25]. The aim of our study was to investigate the influence of haemotherapy with fibrinogen concentrate on myocardial infarct size. There was no difference in infarct size following fibrinogen substitution, indicating that fibrinogen does not affect the extent of acute myocardial infarction. Therefore, the prothrombotic shift after fibrinogen administration may not delay or diminish reperfusion after myocardial ischaemia. These results are strengthened by our finding that neither substitution of a low or high dose fibrinogen influences the speed of arterial thrombogenesis after an experimental vessel lesion compared to control animals.

### 4.2. Fibrinogen and IPC

A potential adverse effect of fibrinogen supplementation cannot only be caused by an aggravation of a thromboembolic event but also by a loss of protective properties. For example, prophylactic treatment with fibrinogen might affect the infarct size reducing effect of IPC. Murry et al. first described the phenomenon of IPC in 1986, where short sublethal periods of myocardial ischaemia and reperfusion before a distinct I/R injury can reduce infarct size up to 75% [11]. Interestingly, IPC causes a significant reduction in platelet activation by diminished fibrinogen activation [12]. In addition, lower extremity IPC by clamping the abdominal aorta for 4 cycles of 5 min ischaemia and reperfusion reduced formation of deep vein thrombosis in rats in an in vivo thrombosis model [26]. Furthermore, brief antecedent myocardial ischaemia even attenuated platelet-mediated thrombosis in damaged and stenotic canine coronary arteries [27]. Taken together, there is clear evidence that conditioning interventions affect the haemostatic/thrombotic homeostasis towards an anti-aggregatory phenotype. In this situation, exogenous administration of fibrinogen might counterbalance the anti-aggregatory effect of IPC, and, thus, its cardioprotective potential. Our results show that IPC initiates a robust infarct size reduction in control animals in our in vivo rat model of myocardial ischaemia and reperfusion. This protective effect of IPC was not affected by previous fibrinogen substitution, indicating that fibrinogen supplementation has no adverse effects with respect to IPC.

## 5. Conclusions

Our findings show that fibrinogen supplementation in a clinically relevant dose using an in vivo rat model has no adverse effects on arterial thrombogenesis, myocardial ischaemia and reperfusion injury, and the cardioprotective potency of IPC.

## Figures and Tables

**Table 1:** Thrombus induction A. femoralis–Blood sampling.

Group	Fibrinogen (mg/dL)	Platelets (*1000/µL)	INR	PTT (s)
Con	200 ± 25	696 ± 100	0.91 ± 0.04	36 ± 22
Fib_low_	261 ± 33 *	787 ± 83	0.84 ± 0.05	34 ± 19
Fib_high_	279 ± 37 *	731 ± 83	0.90 ± 0.08	31 ± 20

Data are mean ± SD. Con = control; INR = international normalised ratio; PTT = prothrombin time, * *p* < 0.05 vs. Con.

**Table 2:** Thrombus induction A. femoralis–Haemodynamic variables.

Group	Heart Rate (bpm)	AOP Mean (mmHg)	Flow A. Femoralis (mL/min)
Con	384 ± 43	110 ± 17	1.26 ± 0.30
Fib_low_	370 ± 37	101 ± 17	1.25 ± 0.31
Fib_high_	369 ± 50	101 ± 25	1.38 ± 0.38

Data are mean ± SD. AOP = aortic blood pressure.

**Table 3:** Area at risk sizes and haemodynamic variables.

**Area at Risk (% of Left Ventricle)**															
Con	Fib_low_			Fib_high_			Con + IPC			Fib_low_ + IPC		Fib_high_ + IPC			
11.3 ± 5.4	16.5 ± 6.4			13.4 ± 4.6			12.0 ± 7.9			12.7 ± 5.4		16.9 ± 8.4			
	**Baseline**			**Washout 3**			**Ischaemia**			**Reperfusion**					
							**15 min**			**30 min**		**120 min**			
**Heart Rate (bpm)**															
Con	358	±	59	355	±	40	334	±	92	392	±	96	394	±	95
Fib_low_	364	±	57	362	±	54	361	±	64	353	±	43	336	±	53
Fib_high_	399	±	35	360	±	87	378	±	42	350	±	64	333	±	64
Con + IPC	413	±	44	386	±	20	391	±	22	380	±	28	366	±	19
Fib_low_ + IPC	410	±	36	349	±	27	364	±	26	376	±	28	321	±	44
Fib_high_ + IPC	397	±	21	362	±	59	352	±	40	361	±	68	326	±	66
**Mean Aortic Pressure (mmHg)**															
Con	95	±	22	94	±	17	87	±	16	78	±	13	71	±	17
Fib_low_	105	±	19	103	±	23	94	±	31	86	±	33	54	±	29 *
Fib_high_	112	±	19	90	±	19	89	±	23	73	±	26 *	51	±	26 *
Con + IPC	123	±	8	92	±	21	90	±	18	85	±	13 *	68	±	18 *
Fib_low_ + IPC	114	±	19	77	±	21 *	77	±	18 *	74	±	24 *	42	±	12 *
Fib_high_ + IPC	114	±	24	88	±	36	95	±	33	75	±	34 *	69	±	25 *

Data are mean ± SD. Con = control; IPC = ischaemic preconditioning; Fib = fibrinogen; AOP = aortic blood pressure. * *p* < 0.05 vs. baseline.
